# Evaluating survival trends over time in patients with metastatic breast cancer and brain metastases: a single center retrospective cohort study

**DOI:** 10.1186/s13058-025-02121-7

**Published:** 2025-10-28

**Authors:** Nira A. Krasnow, Michelle Jayaraj, Mia Salans, Kelsey Kuwahara, Maggie H. Zhou, Samantha Fisch, Lauren Boreta, Steve E. Braunstein, Manish K. Aghi, Hemali Batra-Sharma, Melanie Majure, Jo Chien, Hope S. Rugo, Ramin A. Morshed, Harish N. Vasudevan, Michelle E. Melisko, Laura A. Huppert

**Affiliations:** 1 Department of Medicine, University of California, San Francisco, San Francisco, CA USA; 2 Division of Hematology/Oncology, Department of Medicine, University of California, San Francisco, San Francisco, CA USA; 3 Department of Radiation Oncology, University of California, San Francisco, San Francisco, CA USA; 4 Department of Neurological Surgery, University of California, San Francisco, San Francisco, CA USA; 5 Division of Breast Medical Oncology, City of Hope Comprehensive Cancer Center, Duarte, CA USA

## Abstract

**Background:**

Approximately 20–30% of patients with metastatic breast cancer (MBC) develop brain metastases (BM) over the course of their disease. It is critical to better understand risk factors and survival outcomes in these patients, including those treated in the modern treatment era.

**Methods:**

We identified patients with MBC and BM diagnosed between 1997 and 2024 at our institution. Review of medical records was completed to identify key demographic, clinical, and survival characteristics.

**Results:**

We identified 507 patients with MBC and BMs with the following subtypes: HR+/HER2- (*n* = 184, 36.3%), HER2+ (*n* = 197, 38.9%), and triple negative breast cancer (TNBC; *n* = 126, 24.9%). Median real-world overall survival (rwOS) from the diagnosis of first BM to death was 21.6 months with the longest median rwOS in patients with HER2+ disease (31.0 months) vs. patients with HR+/HER2- (19.6 months) or TNBC (12.8 months) (*p* < 0.001). By date of BM diagnosis 1997–2014 vs. 2015–2024 (divided by ~ 50% of patients in each time period), patients with HER2+ and TNBC lived longer in the more modern cohort compared to prior years (HER2+: 41.2 vs. 26.2 months, *p* = 0.002; TNBC: 14.9 vs. 7.0 *p* = 0.020). There was no statistically significant difference for patients with HR+/HER2- disease (16.5 vs. 21.6, *p* = 0.089). On multivariable analysis, HER2+ disease (HR 0.64, 95% CI 0.50–0.81, *p* < 0.001), BM surgical resection (HR 0.67, 95% CI 0.51–0.87, *p* = 0.002), and BM diagnosis after 2014 (HR 0.77, CI 0.63–0.95, *p* = 0.015) were associated with longer survival. TNBC (HR 1.46, CI 1.12–1.89, *p* = 0.004), having 6–10 BMs at baseline (HR 1.66, CI 1.14–2.42, *p* = 0.009), extracranial MBC (HR 1.34 CI 1.02–1.76, *p* = 0.034) and development of leptomeningeal disease (HR 1.41, CI 1.11–1.80, *p* = 0.005) were associated with shorter survival.

**Conclusion:**

In a cohort of > 500 patients with MBC BMs spanning > 25 years, median rwOS from the diagnosis of first BM was almost two years. Favorable factors included HER2+ disease, BM surgical resection, and diagnosis after 2014. Poor prognostic factors included TNBC, having 6–10 BMs, extracranial MBC, and development of LMD. Patients with HER2+ and TN MBC with BM had improved rwOS in a more modern cohort; this was not seen for HR+/HER2- patients, representing an area of ongoing unmet clinical need.

## Introduction

More than one in four patients with metastatic breast cancer (MBC) will eventually develop brain metastases (BMs), with incidence varying by molecular subtype [1, 2]. As diagnostic strategies improve and treatment options advance, the prevalence of brain metastases appears to be increasing over time as patients survive longer with MBC, so this is an increasing problem [3, 4]. In fact, data has demonstrated that incidence of brain metastases increases with each line of therapy for every breast cancer subtype [5]. Historically, locoregional treatment of BMs via surgical resection and/or irradiation was cornerstone of management, while the use of systemic therapies to treat BMs has been limited by the selective impermeability of the blood brain barrier. However, over the past two decades, a number of novel therapies with improved central nervous system (CNS) penetration have become available, such as antibody drug conjugates and certain targeted agents [6–8]. 

There have been many notable advancements in the treatment of patients with human epidermal growth factor receptor 2-positive (HER2+) MBC with BMs. The HER2-directed antibody drug conjugates trastuzumab emtansine (T-DM1) and trastuzumab deruxtecan (T-DXd) have both recently been shown to have intracranial efficacy and promote BM regression [9–11]. In particular, T-DXd has demonstrated substantial and durable intracranial response responses, even in patients with stable and active brain metastases, with studies supporting improved responses and reduced risk of disease progression with T-DXd compared to T-DM1 [11, 12]. HER2-directed tyrosine kinase inhibitors such as tucatinib, lapatinib, and neratinib represent another class of effective therapies for the treatment of MBC BMs. These lipophilic small molecules are thought to readily penetrate the blood brain barrier, with studies demonstrating improved survival outcomes in patients with MBC BMs that are treated with these therapies [13–15]. 

Novel therapeutics for HER2-negative (HER2-) disease that have proven effective in the context of intracranial disease are more limited. Abemaciclib, a cyclin-dependent kinase (CDK) 4/6 inhibitor, received FDA approval in 2017 for patients with hormone receptor positive (HR+)/HER2- MBC, with subsequent trials supporting its intracranial efficacy [16, 17]. The antibody drug conjugate sacituzumab govitecan has been shown to improve progression free survival and overall survival in patients with triple negative breast cancer (TNBC) and HR+/HER2- MBC when compared to single agent chemotherapy, with evidence of CNS penetration [18–21]. Single agent pembrolizumab, an immune checkpoint inhibitor (ICI), has demonstrated ~20% overall response rate in patients with TNBC, including those with active/symptomatic BMs [1, 19, 22]. Finally, inhibitors of poly (ADP-ribose) polymerase (PARP) have also been evaluated in HER2-negative disease with case reports suggesting clinical benefit in patients with BMs [23, 24]. 

Prior retrospective cohort studies have described the survival of patients with MBC and brain metastases, although many of these studies are older, pre-dating the introduction of many of the therapies outlined above [1, 25, 26]. While more modern studies have been conducted, most have been smaller in size or focused on specific breast cancer subtypes [27, 28]. Thus, it is important to better understand the risk factors and survival characteristics of patients with MBC across breast cancer subtypes, including those treated in the modern therapeutic era. In this retrospective cohort study, we identified a large cohort of patients with MBC and BMs at a single center. Using detailed chart extraction, we evaluated demographic and clinical characteristics, treatment strategies, and survival data in the overall cohort, stratified by molecular subtype and by year of diagnosis, to elucidate how survival outcomes have evolved over the past 25 years.

## Methods

### Study design and objectives

This is a retrospective study of patients with MBC and BMs diagnosed at the University of California San Francisco (UCSF) between 1997 and 2024. The primary objective of this study is to characterize the clinical characteristics and survival outcomes of patients with MBC and brain metastases. Secondary objectives include identifying risk factors associated with poor prognosis.

### Participant identification

Patients were identified in three ways: (1) via a search of the UCSF radiation oncology database for patients with MBC and brain metastases receiving radiation at UCSF; (2) via a search of clinical records for patients with metastatic breast cancer and brain metastases; and (3) via a search of the UCSF pathology database for patients with breast cancer and resected brain metastases. Patients were 18 years of age or older. Patients were excluded during initial screening if they were duplicated or if they did not have a primary diagnosis of breast cancer. After chart abstraction, additional patients were excluded if MBC receptor subtype information was not available (*n* = 7) or if patients had dural or calvarial metastases, and not parenchymal BMs (*n* = 3).

### Chart abstraction

Detailed information including patient demographics and tumor characteristics, treatment history, lab values, clinical outcomes, and survival dates were obtained via manual chart abstraction. Survival data was updated on 3/21/2025.

### Statistics

Data were analyzed using Prism Software (GraphPad; San Diego, CA) and Stata v.18 (StataCorp; College Station, Tx). Descriptive statistics were used to summarize numeric responses as rate of events (%) and median (range) as appropriate. Comparisons between groups were made using the Wilcoxon rank-sum or log-rank (Mantel-Cox) test as appropriate. Survival distributions were estimated by the Kaplan-Meier non-parametric method. Univariable and multivariable analyses of factors associated with median rwOS from diagnosis of BM diagnosis were evaluated using the Cox proportional-hazards model. All variables of interest and with p-value < 0.1 on univariable analysis were included in the final multivariable model. P-value < 0.05 was considered statistically significant.

### Institutional review board statement

This research was approved by the UCSF Institutional Review Board.

## Results

### Patient demographics and clinical characteristics

A cohort of 507 patients at our institution was identified for this study. Patient demographic and clinical characteristics are shown in Table 1. Most patients were female (*n* = 503, 99.2%), white (*n* = 318, 62.7%), and non-Hispanic (*n* = 437, 86.2%). The median age at diagnosis of MBC was 53 years (range: 25–92). Most tumors were ductal histology (*n* = 434, 85.6%). Breast cancer subtypes at MBC diagnosis included HR+/HER2- (*n* = 184, 36.3%), HR+/HER2+ (*n* = 96, 18.9%), HR-negative (HR-)/HER2+ (*n* = 101, 19.9%), and TNBC (*n* = 126, 24.9%). The majority of patients had extracranial disease at the time of BM diagnosis (*n* = 383, 75.5%). The median time from MBC diagnosis to BM diagnosis was 8.1 months (range 0-177.1 months) which differed by receptor subtype with the shortest time to BM in patients with TNBC (2.8 months), followed by HER2+ patients (8.3 months), and HR+/HER2- patients (16.2 months) (*P* < 0.05). At the time of first BM diagnosis, most patients had more than one BM (*n* = 342, 67.5%) with most patients having 2–5 BMs (*n* = 191, 37.7%). Almost 20% of patients (*n* = 95, 18.7%) developed LMD during the course of their disease.

**Table 1:** Patient demographic and clinical characteristics

Characteristic	All patients *N* = 507
Female sex, no. (%)	503 (99.2)
Age at MBC diagnosis, median (range)	53 (25–92)
Race, no. (%)	
White	318 (62.7)
Black	29 (5.7)
Asian	72 (14.2)
Native American or Alaska Native	4 (0.8)
Native Hawaiian or Pacific Islander	7 (1.4)
Other	66 (13.0)
Unknown	11 (2.2)
Ethnicity, no. (%)	
Hispanic	49 (9.7)
Non-Hispanic	437 (86.2)
Unknown	21 (4.1)
Tumor histology, no. (%)	
Ductal	434 (85.6)
Lobular	25 (4.9)
Mixed ductal/lobular	10 (2.0)
Other	9 (1.8)
Unknown	29 (5.7)
MBC subtype, no. (%)	
HR+/HER2-	184 (36.3)
HER2+	
HR+/HER2+	96 (18.9)
HR-/HER2+	101 (19.9)
TNBC	126 (24.9)
Extracranial MBC at time of BM diagnosis	383 (75.5)
BM as initial site of MBC	133 (26.2)
# of BM at first BM diagnosis	
1	156 (30.8)
2–5	191 (37.7)
6–10	48 (9.5)
>10	103 (20.3)
Unknown	9 (1.8)

Abbreviations: Brain metastases (BM); hormone receptor (HR); human epidermal growth factor receptor 2 (HER2); metastatic breast cancer (MBC); number (no.); triple negative breast cancer (TNBC).

### Systemic therapy in the metastatic setting prior to and after the diagnosis of BMs

Systemic therapies in the metastatic setting received before and after the diagnosis of BM are summarized in Table 2. Prior to the diagnosis of BMs, across all breast cancer subtypes patients received a median of 1 total line of therapy for MBC (range 0–11), including a median of 1 prior line of chemotherapy (range 0–10) and 0 prior lines of endocrine therapy (range 0–7). There was no significant difference in number of total treatment lines prior to BM diagnosis when comparing patients diagnosed before 2014 to those after 2014 overall (median 1 vs. 1, *p* = 0.673) or by MBC subtype (HR+/HER2-: 2 vs. 2, *p* = 0.440; HER2+: 1 vs. 1, *p* = 0.195; TNBC: 1 vs. 0, *p* = 0.518). After the diagnosis of BMs, patients received a median of 2 lines of systemic therapy (range 0–16), including a median of 1 line of chemotherapy (range 0–9) and 0 lines of endocrine therapy (range 0–5). Most patients received CNS-directed radiation for their BM (*n* = 478, 95.2%) in the form of stereotactic radiosurgery (*n* = 418, 83.3%) and/or whole brain radiation (*n* = 219, 43.6%). Nearly one third of patients (*n* = 152, 30.0%) underwent BM surgical resection. Many patients were treated with novel CNS-penetrant therapies following BM diagnosis such as HER2-directed TKIs (*n* = 97, 19.6%), abemaciclib (*n* = 14, 2.8%), T-DM1 (*n* = 43, 8.7%), T-DXd (*n* = 51, 10.3%), sacituzumab govitecan (*n* = 29, 5.9%), and/or checkpoint inhibitors (*n* = 34, 6.9%). Rates of use of these therapies prior to BM diagnosis are outlined in Table 2.

**Table 2:** Systemic and locoregional treatments in the metastatic setting prior to and after the diagnosis of BMs

	All patients	HR+/HER2-		HER2+	TNBC	
Systemic Therapies for MBC Before BM diagnosis						
Lines chemotherapy, med (range)	1 (0–10)		1 (0–7)	1 (0–10)		0 (0–8)
Lines ET, med (range)	0 (0–7)		1 (0–7)	0 (0–4)		0 (0–1)
Lines total therapy, med (range)	1 (0–11)		2 (0–11)	1 (0–10)		0 (0–8)
CNS-active therapies of interest, no. (%)^a^						
HER2-directed TKIs	29 (5.9)		0 (0.0)	28 (14.4)		1 (0.8)
Abemaciclib	6 (1.2)		5 (2.8)	1 (0.5)		0 (0.0)
T-DM1	20 (4.0)		1 (0.6)	18 (9.3)		1 (0.8)
T-DXd	11 (2.2)		5 (2.8)	6 (3.1)		0 (0.0)
Sacituzumab govitecan	6 (1.2)		0 (0.0)	0 (0.0)		6 (4.9)
Checkpoint inhibitors	17 (3.4)		6 (3.4)	1 (0.5)		10 (8.2)
**Systemic Therapies for MBC After BM diagnosis**						
Lines chemotherapy, med (range)	1 (0–9)		1 (0–9)	1 (0–8)		1 (0–7)
Lines ET, med (range)	0 (0–5)		0 (0–5)	0 (0–3)		0 (0–2)
Lines total therapy, med (range)	2 (0–16)		2 (0–16)	2 (0–10)		1 (0–8)
CNS-active therapies of interest, no. (%)^a^						
HER2-directed TKIs	97 (19.6)		4 (2.2)	92 (47.4)		1 (0.8)
Abemaciclib	14 (2.8)		14 (7.8)	0 (0.0)		0 (0.0)
T-DM1	43 (8.7)		5 (2.8)	37 (19.1)		1 (0.8)
T-DXd	51 (10.3)		17 (9.5)	27 (13.9)		7 (5.7)
Sacituzumab govitecan	29 (5.9)		14 (7.8)	1 (0.5)		14 (11.5)
Checkpoint inhibitors	34 (6.9)		12 (6.7)	4 (2.1)		18 (14.8)
Radiation therapy, no. (%)^b^						
Any CNS-directed radiation	478 (95.2)		172 (94.0)	189 (96.9)		117 (94.4)
SRS	418 (83.3)		156 (85.3)	162 (83.1)		100 (80.7)
WBRT	219 (43.6)		75 (41.0)	99 (50.8)		45 (36.3)
Surgical resection of BM, no. (%)^c^	152 (30.0)		34 (18.5)	74 (37.8)		44 (34.9)

Abbreviations: Brain metastasis (BM); central nervous system (CNS); endocrine therapy (ET); human epidermal growth factor receptor 2 (HER2); median (med); stereotactic radiosurgery (SRS); trastuzumab deruxtecan (T-DXd); trastuzumab emtansine (T-DM1); tyrosine kinase inhibitor (TKI); whole-brain radiation therapy (WBRT).

^a^ Systemic treatment records were available for *n* = 495 patients: *n* = 179 HR+/HER2-, *n* = 194 HER2+, *n* = 122 TNBC

^b^ Radiation records were available for *n* = 502 patients: *n* = 183 HR+/HER2-, *n* = 195 HER2+, *n* = 124 TNBC

^c^ Surgical records were available for *n* = 506 patients: *n* = 184 HR+/HER2-, *n* = 196 HER2+, *n* = 126 TNBC

### Overall survival from diagnosis of BMs to death and risk factors associated

**Fig. 1:**
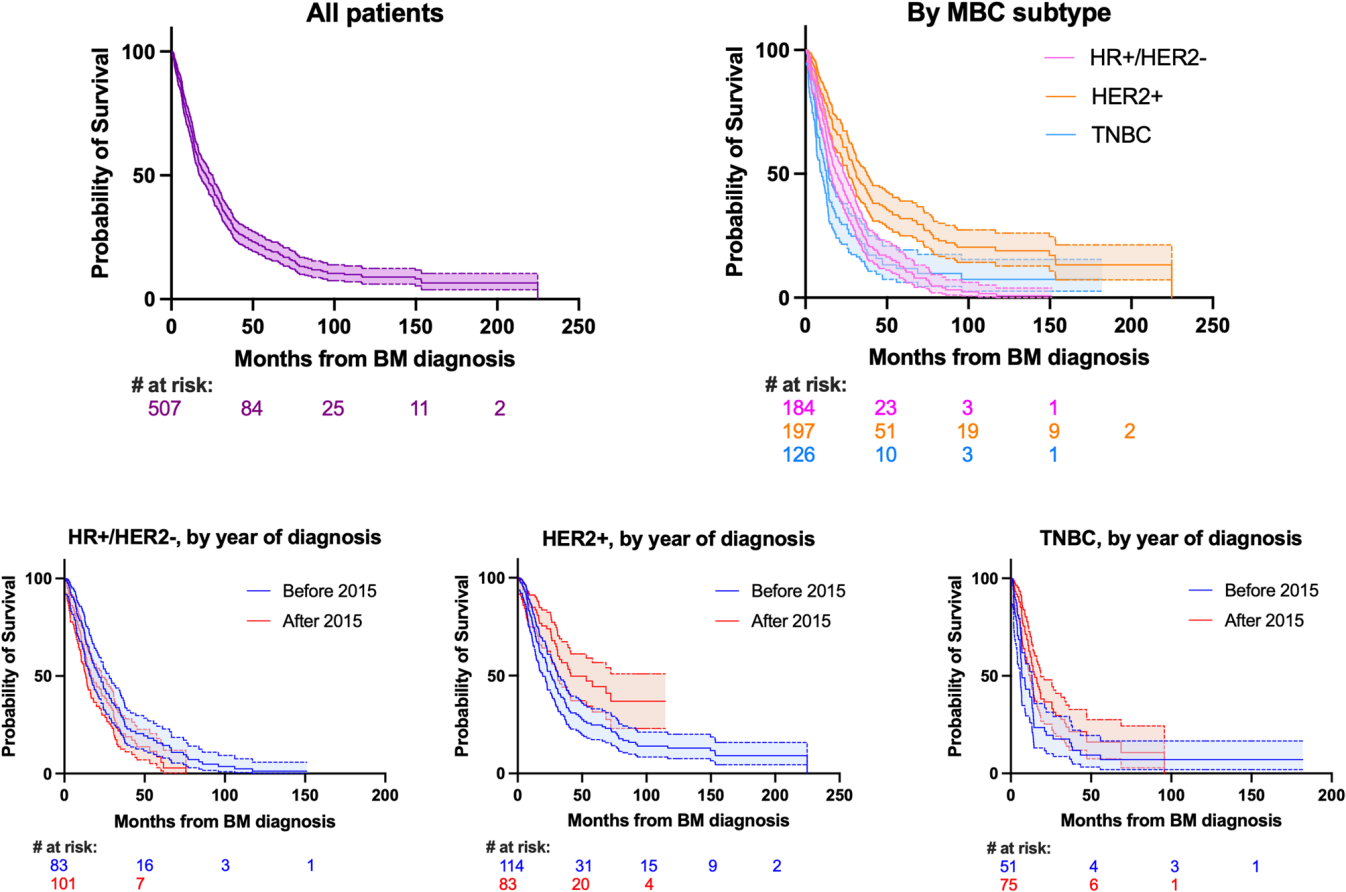
Overall survival from diagnosis of brain metastases to death. Abbreviations: Brain metastasis (BM); hormone receptor (HR); human epidermal growth factor receptor 2 (HER2); metastatic breast cancer (MBC); triple negative breast cancer (TNBC)

Kaplan-Meier curves for survival data from the diagnosis of BMs to death are shown in Fig. 1 and data is summarized in Table 3. Median rwOS, in all patients, from the diagnosis of BMs to death was 21.6 months (interquartile range [IQR] 9.3–45.3 months). Median rwOS from BM diagnosis to death varied by subtype, with patients with HER2+ MBC living longer than those with HR+/HER2- or TN (triple negative) MBC (median 31.0 months vs. 19.6 months and 12.8 months, log-rank *p* < 0.001 for both comparisons). Patients with HER2+ and TN MBC who were diagnosed with BM after 2014 lived longer than patients with HER2+ and TN MBC who were diagnosed 2014 or earlier, respectively (HER2+ median: 41.2 months vs. 26.2 months, *p* = 0.002; TNBC median: 14.9 months vs. 7.0 months, *p* = 0.020). Conversely, there was no significant difference in survival by year of BM diagnosis 2015–2024 vs. 1997–2014 for patients with HR+/HER2- MBC (median 16.5 months vs. 21.6 months, *p* = 0.089).

**Table 3:** Overall survival from diagnosis of brain metastases to death

	Median OS in months (IQR)	*p*-value (log-rank)
All patients (*n* = 507)	21.6 (9.3–45.3)	---
**By MBC molecular subtype**		
HR+/HER2- (*n* = 184)	19.6 (10.0-35.6)	< 0.001^a*^
HER2+ (*n* = 197)	31.0 (14.3–78.3)	---
TNBC (*n* = 126)	12.8 (6.1–28.0)	< 0.001^a*^
**By year of BM diagnosis**		
HR+/HER2-		0.089
1997–2014 (*n* = 83)	21.6 (11.3–37.7)	
2015–2024 (*n* = 101)	16.5 (8.9–32.7)	
HER2+		0.002^*^
1997–2014 (*n* = 114)	26.2 (11.9–57.2)	
2015–2024 (*n* = 83)	41.2 (22.5-NR)	
TNBC		0.020^*^
1997–2014 (*n* = 51)	7.0 (3.9–14.4)	
2015–2024 (*n* = 75)	14.9 (8.7–34.4)	
**By number of BMs**		
1 (*n* = 156)	28.8 (10.8–65.4)	---
2–5 (*n* = 191)	21.8 (9.3–47.3)	0.102^b^
6–10 (*n* = 48)	13.1 (5.0-26.5)	0.017^b*^
>10 (*n* = 103)	18.4 (10.5–32.5)	0.122^b^

Abbreviations: Brain metastases (BM); hormone receptor (HR); human epidermal growth factor receptor 2 (HER2); interquartile range (IQR); metastatic breast cancer (MBC); not reached (NR); number (no.); overall survival (OS); triple negative breast cancer (TNBC).

^a^ Compared to HER2+ subtype

^b^ Compared to numerically preceding category (1 vs. 2–5 BMs; 2–5 vs. 6–10 BMs; 6–10 vs. >10 BMs)

^*^ Indicates significance at *p* < 0.05

Results of univariable and multivariable analyses of factors associated with median rwOS from time of BM diagnosis to death are shown in Table 4. On multivariate analysis, HER2+ status (HR 0.64, *p* < 0.001), BM diagnosis after 2014 (HR 0.77, *p* = 0.015), and surgical resection of BM (HR 0.67, *p* = 0.002) were associated with improved median rwOS. Extracranial metastatic sites at time of BM diagnosis (HR 1.34, *p* = 0.034), development of leptomeningeal disease (HR 1.41 *p* = 0.005), TNBC status (HR 1.46, *p* = 0.004), and having 6–10 BMs (HR 1.66, *p* = 0.009) were associated with worse rwOS.

**Table 4:** Factors associated with overall survival from the diagnosis of brain metastases to death in univariable and multivariable analysis

	Univariate analysis			Multivariate analysis^a^		
	HR	95% CI	*p*-value	HR	95% CI	*p*-value
Age at MBC diagnosis	1.01	1.00-1.01	0.164			
Race						
White	Ref	-	-			
Asian	0.97	0.72–1.29	0.815			
Black	1.00	0.64–1.56	0.996			
Other	0.81	0.61–1.08	0.156			
Year of BM diagnosis						
1997–2014	Ref	-	-	Ref	-	-
2015–2024	0.88	0.72–1.07	0.193	0.77	0.63–0.95	0.015^*^
MBC subtype						
HR+/HER2-	Ref	-	-	Ref	-	-
HER2+	0.55	0.44–0.69	< 0.001^*^	0.64	0.50–0.81	< 0.001^*^
TNBC	1.20	0.94–1.53	0.154	1.46	1.12–1.89	0.004^*^
Extracranial MBC at time of BM diagnosis	1.64	1.30–2.08	< 0.001^*^	1.34	1.02–1.76	0.034^*^
Number of BMs						
1	Ref	-	-	Ref	-	-
2–5	1.23	0.96–1.56	0.097	1.21	0.94–1.56	0.145
6–10	1.92	1.34–2.77	< 0.001^*^	1.66	1.14–2.42	0.009^*^
>10	1.46	1.10–1.92	0.008^*^	1.27	0.94–1.70	0.116
CNS-directed radiation	0.74	0.46–1.19	0.210	0.70	0.43–1.14	0.149
BM surgical resection	0.55	0.44–0.69	< 0.001^*^	0.67	0.51–0.87	0.002^*^
Diagnosis of LMD	1.40	1.10–1.77	0.005^*^	1.41	1.11–1.80	0.005^*^

Abbreviations: Brain metastasis (BM); confidence interval (CI); diagnosis (dx); hormone receptor (HR); human epidermal growth factor receptor 2 (HER2); leptomeningeal disease (LMD); metastatic breast cancer (MBC); stereotactic radiosurgery (SRS); triple negative breast cancer (TNBC); whole brain radiation therapy (WBRT).

^**a**^All variables of interest and with p-value < 0.1 on univariable analysis were included in multivariable analysis

^*^ Indicates significance at *p* < 0.05

## Discussion

In this large single-center retrospective cohort study we retrospectively reviewed over 500 patients with MBC BMs spanning over 25 years of treatment, elucidating updated treatment characteristics, risk and protective factors, and survival trends in this population.

In our cohort, median rwOS among all patients was 22 months, with HER2+ patients surviving 31 months, which was longer than HR+/HER2- and TNBC patients who survived 20 months and 13 months respectively. Prior studies have shown similar trends but comparatively shorter survival times, with all patients surviving a median of 7–10 months from BM diagnosis, HER2+ patients surviving 10–26 months, HR+/HER2- patients surviving 6–14 months, and TNBC patients surviving 4–7 months [1, 28–34]. The reasoning behind the discrepancy between our results and prior studies is likely multifactorial, in part because some patients in our cohort were treated more recently (through 2024); thus, many patients received novel CNS-penetrant therapies and modern radiation techniques that were not part of routine clinical care in older cohorts. In addition, our cohort was younger (median age 53), patients were less heavily pre-treated (median 1 line of prior systemic therapy), and patients received treatment at a tertiary academic center with access to highly specialized care and clinical trials. Indeed, one study that examined survival among HER2+ MBC patients with BMs who were similarly treated at a highly specialized cancer center in the US in a more recent timeframe (through 2022) found survival to be ~26 months, which is more similar to our results [28]. 

We found that patients with HER2+ and TN MBC who were diagnosed after 2014 lived significantly longer than patients of the same subtype who were diagnosed 2014 or earlier. Conversely, we found no difference in survival by year of diagnosis for patients with HR+/HER2- MBC. Witzel et al. performed a similar study comparing survival among patients with MBC BMs diagnosed between 2000–2009, and 2010–2015. They found that median survival decreased over time when comparing the older and more modern cohorts (7.6 months vs. 5.8 months, *p* < 0.001), though this was not stratified by molecular subtype [30]. Given diagnostics and locoregional treatment advances for BMs are typically subtype agnostic, the improvement in survival among patients with HER2+ and TN MBC observed in our study is likely due, at least in part, to the advances in systemic treatments for these subtypes. This highlights the importance of the ongoing inclusion of patients with MBC BMs in therapeutic clinical trials, with our data demonstrating an impact on real-world survival in just the past ten years. Concurrently, our results suggest a potential area of unmet clinical need for new systemic treatment strategies for patients with HR+/HER2- MBC with BMs. It is worth noting that while many novel therapeutics for MBC have been shown to have intracranial efficacy, it remains possible that the survival benefit is due to improved treatment of extracranial disease, rather than improved CNS disease control.

On multivariate analysis of factors associated with improved survival, similar to prior studies, we found that surgical resection of BMs and HER2+ molecular subtype were protective, in addition to diagnosis after 2014 [1, 25, 35]. It is worth noting that the benefit of surgical resection may be serving as a proxy for performance status, which has been shown in prior studies to be strongly associated with survival in MBC patients with BMs [36]. Conversely, we found that extracranial MBC, development of LMD, TNBC status, and having 6–10 MBs were associated with worse median rwOS, which have also been demonstrated previously [1, 25, 29, 35, 37]. Interestingly, the presence of more than 10 BMs was not associated with worse survival on multivariate analysis. This could be related to smaller size of lesions (based on review, many of these patients had small, punctate lesions), which has been previously shown to influence survival, or the analysis may have been limited by smaller sample size in this group [35]. 

In our study, the median time between MBC diagnosis and BM diagnosis was 8 months and patients received a median of one line of systemic treatment for MBC prior to diagnosis of BMs. The median time from MBC to BM diagnosis differed by tumor biology with the longest time to BM diagnosis seen in patients with HR+/HER2- MBC and shortest time to BM diagnosis in patients with TNBC, confirming trends in prior studies [26, 38]. This is shorter than what has been previously described and what we have observed clinically, particularly for HR+/HER2- patients, and likely indicates some amount of selection bias in our cohort for patients who were diagnosed with BMs earlier in their disease course [5, 26]. Indeed, patients who had BMs as their first site of MBC comprised a substantial fraction (26%) of our cohort. However, total treatment lines for MBC prior to BM diagnosis were similar when comparing patients diagnosed 2014 or earlier and those after 2014, both overall and by MBC subtype; thus, lead time bias is unlikely to explain the improvement in survival that we observed in HER2+ and TNBC patients in the more modern treatment cohort.

There were high numbers of patients who underwent surgical resection (30% of our cohort), which may be due to overrepresentation of patients identified via pathology reports, rather than radiation or clinical records, though these rates are similar to prior survival analyses [28, 30]. Patients with HER2+ disease and TNBC were also over-represented in our cohort compared to the prevalence in the overall MBC population, though this likely reflects a biologically-driven propensity for CNS disease development in HR-negative tumors, which has been previously described [1, 39, 40]. Interestingly, our cohort contained a low percentage of patients with lobular (4.9%) or mixed lobular/ductal MBC (2.0%), which is unexpected as lobular tumors comprise up to 15% of new breast cancer diagnoses with studies suggesting similar rates of CNS metastases when compared to ductal tumors [41, 42]. 

Our study had certain limitations. First, this was a retrospective study conducted at a single academic center with high rates of tertiary referrals for radiation and neurosurgical intervention, as well as ongoing clinical trials. Thus, generalizability of these results may be limited. Second, we did not have a control arm of patients with MBC without BMs, which would allow us to identify risk factors for development of BMs as well as incidence of BMs; this could be considered as a future analysis. Third, current guidelines do not mandate brain imaging at the time of diagnosis of locally advanced or MBC, thus there is intrinsic inaccuracy in the date of BM diagnosis, though this would be more likely to underestimate, rather than overestimate real-world survival in our cohort. Fourth, there are additional variables that can be considered in future survival analyses, including inflammatory breast cancer status, programmed cell death protein 1 (PD-1) status, and performance status. Finally, while we suspect that the use of CNS-penetrant novel therapeutics contributed to the improvement in survival that was observed in patients with HER2+ and TN MBC, numbers of patients were too small to test this association. It would also be interesting to evaluate if increased use of CNS-penetrant therapies prior to the development of BMs delays time to development of CNS disease. Larger studies will be needed to evaluate the associations between the use of novel CNS-penetrant therapies, time to development of CNS disease, and real-world overall survival.

In conclusion, in a cohort of over 500 patients with MBC and BMs treated at a single center spanning over 25 years, median rwOS from the diagnosis of first BM to death was almost two years, with longer survival in patients with HER2+ BM (31 months) compared to those with HR+/HER2- (20 months) and TNBC patients (13 months) respectively. Additionally, patients with HER2+ and TN MBC with BMs had improved rwOS in a more modern cohort (2015–2024) compared to those diagnosed earlier (1997–2014). There was no difference in rwOS in these time periods for patients with HR+/HER2- MBC, suggesting an area of ongoing unmet clinical need, and a potential population of choice for ongoing development for novel CNS-penetrant agents.

## Data Availability

The datasets generated during the current study are not publicly available in order to protect patient privacy.

## Abbreviations

BM: Brain metastasis
CNS: Central nervous system
ET: Endocrine therapy
HER2: Human epidermal growth factor receptor 2
HR: Hormone receptor
med: Median
SRS: Stereotactic radiosurgery
T-DXd: Trastuzumab deruxtecan
T-DM1: Trastuzumab emtansine
TKI: Tyrosine kinase inhibitor
TNBC: Triple negative breast cancer
WBRT: Whole brain radiation therapy
